# Influence of Chromium-Cobalt-Molybdenum Alloy (ASTM F75) on Bone Ingrowth in an Experimental Animal Model

**DOI:** 10.3390/jfb9010002

**Published:** 2017-12-26

**Authors:** Jésica Zuchuat, Marcelo Berli, Ysaí Maldonado, Oscar Decco

**Affiliations:** 1 Bioimplants Laboratory, Faculty of Engineering, National University of Entre Rios, Oro Verde, Entre Rios E3100, Argentina; odecco@bioingenieria.edu.ar; 2 Computational Biomechanics Group, Faculty of Engineering, National University of Entre Rios, Oro Verde, Entre Rios E3100, Argentina; mberli@bioingenieria.edu.ar; 3 Imaging Service, Sanatorio Adventista Del Plata, 25 De Mayo 255, Villa Libertador General San Martín, Entre Ríos E3103, Argentina; ysai.maldonado@sanatorioadventista.com.ar

**Keywords:** Cr-Co-Mo alloy, barrier membrane, bone augmentation, plasma exudate, bone density

## Abstract

Cr-Co-Mo (ASTM F75) alloy has been used in the medical environment, but its use as a rigid barrier membrane for supporting bone augmentation therapies has not been extensively investigated. In the present study, Cr-Co-Mo membranes of different heights were placed in New Zealand white, male rabbit tibiae to assess the quality and volume of new bone formation, without the use of additional factors. Animals were euthanized at 20, 30, 40, and 60 days. Bone formation was observed in all of the cases, although the tibiae implanted with the standard membranes reached an augmentation of bone volume that agreed with the density values over the timecourse. In all cases, plasmatic exudate was found under the membrane and in contact with the new bone. Histological analysis indicated the presence of a large number of chondroblasts adjacent to the inner membrane surface in the first stages, and osteoblasts and osteocytes were observed under them. The bone formation was appositional. The Cr-Co-Mo alloy provides a scaffold with an adequate microenvironment for vertical bone volume augmentation, and the physical dimensions and disposition of the membrane itself influence the new bone formation.

## 1. Introduction

Bone resorption occurs in a wide variety of clinical situations, such as trauma, injury, bone illness, or hormonal disorders. This decrease, either in bone volume or in bone quality, can produce aesthetic and functional complications, depending on the defect location and the function of the involved bone, that directly affect the patient’s activities and increases morbidity.

During the last few years, remarkable progress in the development of new orthopaedic techniques (i.e., distraction osteogenesis, bone augmentation through the application of rhBMP-2 and genetic therapies) and a great number of surgical procedures has been created to support and improve bone reconstruction outcomes [1]. Although those techniques have significantly transformed reconstructive surgery and improved clinical results, currently they have some limitations, such as the shortage of required materials, since autologous bone grafts are employed as the standard against which the rest of the methods are compared [2,3]. Autologous bone combines all of the properties of a graft material, providing a scaffold for the inner growth of osteoprogenitor cells (osteoconductivity) and stimulating stem cell proliferation and differentiation in osteogenic cell lines (osteoinductivity) to help maintain the viable cells that form new bone tissue (osteogenicity) [3,4]. However, the amount of autologous bone available from a patient is limited and requires an additional surgical procedure to harvest it, which increases the morbidity of the donor site and may cause chronic pain or dysesthesia in the harvest site [5].

Recent advances in bone tissue engineering have given surgeons new treatment options in order to restore the form and function of the involved tissue [4]. Research and development of different technologies include three-dimensional (3D) printing, sputtering coatings, and electro-spinning techniques for the development of resorbable and non-resorbable materials. These materials are combined with growth factors, bone morphogenetic proteins (BMPs), or other elements or substances, which act as carriers. Certain substances regulate the actions of osteoprogenitor cells at the target site and control the degradation and physical resorption rates in the host environment, so the materials are replaced by the regenerated bone. These new techniques and new computational models that are capable of predicting bone evolution and bone remodelling are intended to overcome the problems found in the previous models. They are seen as promising approaches for the prediction of bone growth and for faster and easier methods of tissue regeneration and repair.

Masquelet et al. (1996) [6] described for the first time a technique of two stages with the aim to take advantage of the immune response against the implantation of foreign materials for the reconstruction of critical bone defects. The Masquelet technique is based on the temporary placement of polymethylmethacrylate (PMMA) membranes. They are fixed to cover the site of the defect and create a biologically active tissue, called an “inducted membrane”, which is highly vascularized and rich in growth factors, such as vascular endothelial growth factor (VEGF) and transforming growth factor beta (TGF-β1) [7,8,9]. After approximately four or eight weeks, a bone substitute, generally an autologous bone graft that includes all of the possible limitations mentioned above, is placed. Since the first study, the Masquelet technique has been widely used and modified with the development of new technologies to solve massive bone defects, while maintaining its clinical efficacy [10,11,12]. In a similar way, in the last few decades, the rigid, osteoconductive, and non-resorbable barrier membranes are used as part of regenerative treatments, and bone augmentation therapies have been thoroughly researched [13] as additional methods to improve osteoinduction and cell differentiation mechanisms [14,15,16,17,18,19]. Membranes contribute to the creation of a favourable microenvironment in the lesion site. There is evidence that those membranes could prevent the migration of non-desirable cells and allow for the migration of osteoprogenitor cells, guaranteeing the formation of new bone tissue and avoiding fibrous tissue proliferation.

The Cr-Co-Mo alloy (ASTM F75) was one of the first Cobalt-based alloys that was used as a biomaterial and gained popularity for its use as orthopaedic implant material, especially in total joint replacements and in odontology [20]. It is currently used because of its biocompatibility and good mechanical properties [21,22]. Previous studies done by this research group have concluded that membranes that are generated by the cast manufacturing method of Cr-Co-Mo alloy have as good performance for bone formation when used in combination with synthetic bone substitutes as autogenous substances do when they were tested in an animal model [14,15]. Additionally, it has been observed that the implantation of the Cr-Co-Mo alloy itself in control animals has the same results as those in the previous case, although in a longer time. It is believed that the alloy has promoted and induced osteoblast migration to the site of the implantation, influencing their proliferation following bone augmentation and assuming a centripetal pattern of growth.

The aim of the present study was to assess the induction of the Cr-Co-Mo alloy on the bone formation rate though the implantation of membranes of two heights in rabbit tibia. We assessed the pattern of growth of new bone in a longitudinal study to determine if the differentiation of the mesenchymal stem cells into osteoblast cells is induced by the membrane itself or another origin, or both. The results were assessed and compared microscopically, densitometrically, radiologically, and in computed tomography (CT) images.

## 2. Results

### 2.1. Macroscopic Analysis

**Figure 1:**
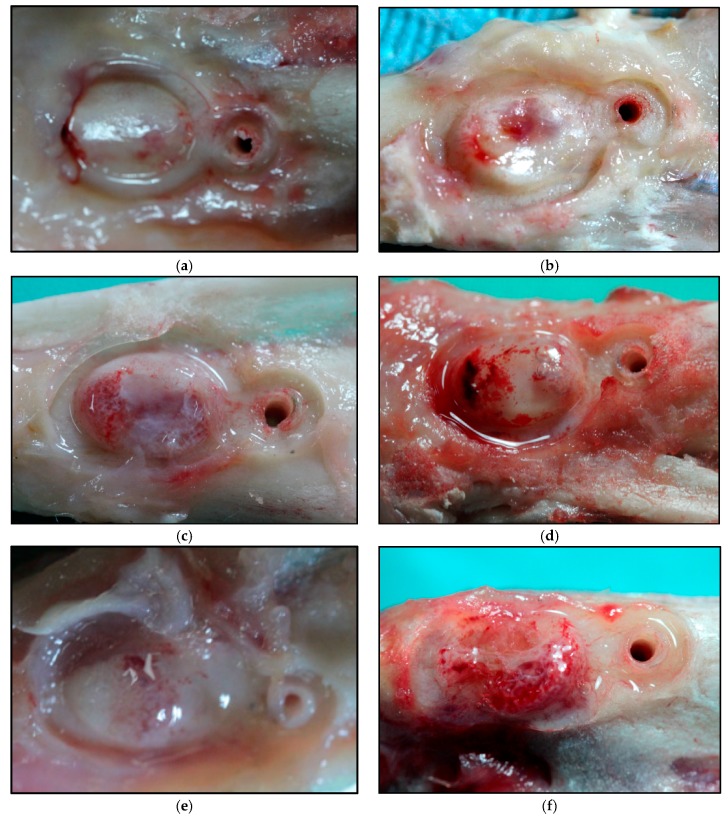

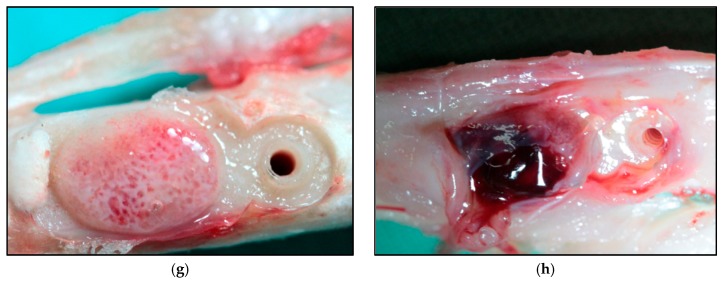
Macroscopic images of the augmented bone volume at: 20 days for SM (**a**) and HM (**b**); 30 days for SM (**c**) and HM (**d**); 40 days for SM (**e**) and HM (**f**); 60 days for SM (**g**) and HM (**h**). SM: Tibia implanted with standard membrane and HM: Tibia implanted with high membrane.

After the sacrifice of each of the experimental animals, bone augmentation was observed (Figure 1) in all of them. We even in some cases observed bone augmentation over the Co-Cr membrane, in its surroundings, and at the micro-screw.

### 2.2. Tomographic Analysis

**Figure 2:**
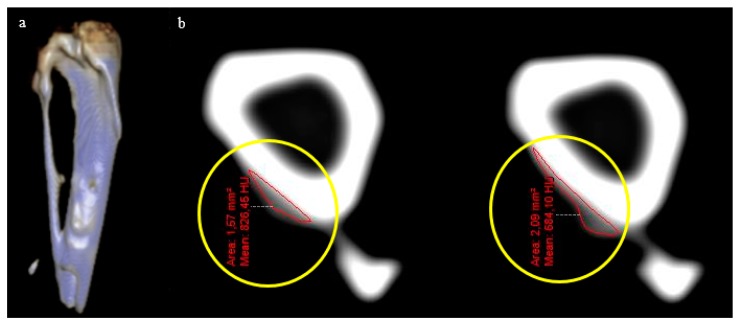
(**a**) Three-dimensional (3D) reconstruction of the rabbit tibia with the augmented bone volume. (**b**) Transversal cuts (1 mm) from which the augmented volumes were quantified (marked in the images by yellow circles).

Due to the longitudinal character of the present study, the changes in bone volume was non-uniform in most of the cases, which made its quantification difficult. The CT images were used to obtain the 3D reconstruction and to assess the volume obtained in each stage of the study (Figure 2). The augmented volumes are shown in Table 1.

### 2.3. Histological Analysis

The bone biopsies from the day of surgery were analysed to describe the remodelling activity in the zones surrounding the location of the membrane. The analysis was performed by the protocol described in Section 2.3. The number of cells was quantified at 40×. A mean number of 117.5 osteocytes in the tibiae implanted with the standard membranes and a mean of 120.0 osteocytes in the tibiae implanted with the high membranes were found. No osteoblasts or osteoclasts were noted in any of the cases. There were no apparent differences in osteocyte populations between tibiae or animals.

**Figure 3:**
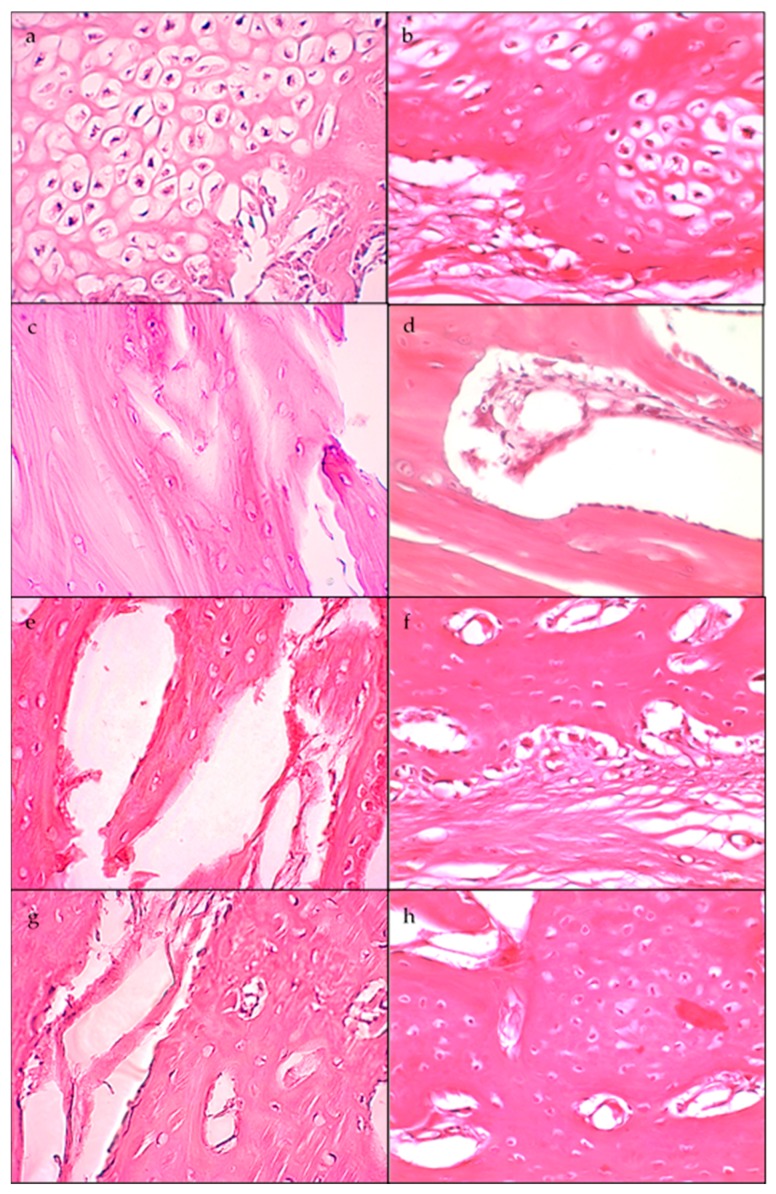
Histological slices (40×) corresponding to: 20 days of SM (**a**) and HM (**b**), where a greater number of osteoblasts closer to the surface of the bone can be observed; 30 days of SM (**c**) and HM (**d**); 40 days of SM (**e**) and HM (**f**); 60 days of SM (**g**) and HM (**h**). SM: Tibia implanted with standard membrane and HM: Tibia implanted with high membrane.

For the histological analysis, the bone samples were cut in longitudinal sections on the largest radius of the augment. The samples were prepared to analyse histological components with the aim to elucidate the mechanism of bone formation (Figure 3).

The cell quantification of samples from the augmented region is shown in Table 2.

Chondroblasts were not quantified, but a large number of them were observed, especially in the augments of animals sacrificed at that were 20 and 30 days of study, constituting an osteocartilaginous tissue. Laminar and osteoid bone was found in all of the augmented bones of the animals that were sacrificed at 40 and 60 days postoperatively.

### 2.4. Densitometric Analysis

The density differences between samples with standard and high membranes were analysed over the timecourse and are shown in Table 3. Values are also presented as ratio between the ROIint (Region of Interest of the bone formation) and ROIref (Region of Interest of reference) throughout the study.

**Figure 4:**
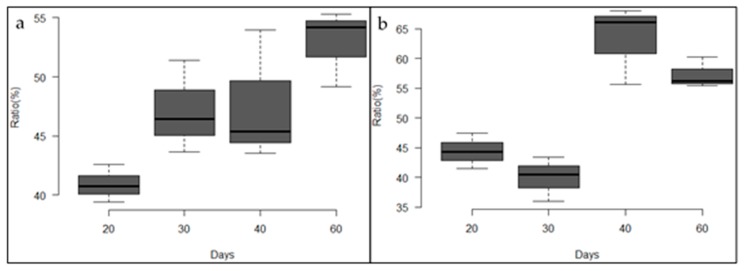
(**a**) Box plot of the density ratio of tibiae implanted with the standard membrane. (**b**) Box plot of the density ratio of tibiae implanted with the high membrane.

In Figure 4a, it can be observed that the mean ratio of density values for the tibiae implanted with the standard membrane increased progressively during the experimental period. In Figure 4b, the box plot shows the values of the tibiae implanted with the high membrane.

## 3. Discussion

Most of the studies concerning vertical bone augmentation from non-critical bone defects involve the use of different techniques and materials, which are generally combined with natural, autologous substrates. Grafts, growth factors, mesenchymal stem cells, and bone morphogenetic proteins, among others [14,15,23,24], are used to obtain successful results in animals [25] and humans [26]. In complex clinical situations, it is unclear if it is possible to augment the bone beyond the bone surface, and this remains an aspect to be investigated. Some studies have shown that it is possible [27,28]. Those defects in which the membrane is not supported adequately by local bone walls, as in the sites for localized vertical augmentation of the bone crest are more difficult to reach. To overcome this obstacle, resorbable membranes have been used. The material degradation rate can significantly alter bone formation [29,30]. When the membranes are exposed to inflammatory reactions of the adjacent tissue, the enzymatic activity of macrophages and neutrophils causes early membrane degradation, affecting its structural integrity and resulting in a decrease in the barrier function. Other studies [14,15,29] have centred on the use of rigid, non-resorbable membranes and fixation and support devices, such as microscrews and microplates. The creation and maintenance of a space between the membrane and the bone surface is essential to reach a successful bone augmentation procedure, and those studies demonstrate that it is possible for bone to form in an intramembranous way beyond the bone surface.

In the present work, rigid Cr-Co-Mo membranes were used to generate a space. No additional variables were used, working only on the subjacent bone surface. In all of the cases, the presence of an exudate over the inner surfaces of the membranes and over the newly formed bone was observed. Membranes were implanted under the surgical incision site, and they were microfixed to the bone surface, producing an initial accumulation of neutrophils at the site, followed by a macrophage infiltration, in which the phagocytes damaged the tissue, triggering the release of growth factors, such as PDGF, TGF-α, and TGF-β1. The plasmatic exudate that is produced by the initial cicatrisation process is fundamentally composed of growth factors and proteins, which have a stimulatory effect on the cellular activity during the new bone formation period. In addition, insulin-like growth factors are produced. They have a known stimulator influence on the osteoblastic activity [31]. These findings coincide with those reported by Krishnan, V. and Davidovitch, Z. (2015) [32], who stated that the vasodilation of the area above a lesion is conducive to the generation of a plasma exudate and the migration of leukocytes out of blood vessels. Leukocytes occupy the extravascular space in compromised tissues and release cytokines, and growth factors to stimulate bone remodelling. On bone formation other hand, Cassell O.C.S et al. (2006) [33] have developed a model, which allowed for the generation of new tissue in the absence of an added matrix, and under these circumstances, a fibrin-rich plasma exudate fills the chamber in the first 24 h and acts as the scaffold.

The use of non-resorbable membranes guarantees a sufficient rigidity in order to avoid wall collapse and to allow for the growth of new bone in the inner space [30]. The surface characteristics of the membranes that are used in this study give it properties that favour the bone formation process. The sandblasting with aluminium particles leads to a roughness of 180 µm, within the optimal range established by Yang, S.L.S. (2015) [34], who expressed that uniform patterns (100 and 500 µm between consecutive indentations) of peaks and valleys provide a larger surface area and are more conductive surfaces for the anchorage and securing the tissues. This allowed a progressive augmentation of the newly formed bone volumes with time in the tibiae implanted with the standard membrane. However, for the high membrane, an initial augmentation (until 30 days after surgery) was observed, and then the augmentation decreased progressively towards the end of the study (Table 1). The reason that the higher membranes led to a slower bone formation can be explained by the potential that is generated between the surface charges of both the cortical bone (which has an electronegativity in the range of −5 to +5 mV [35]) and those created by the sandblasting that formed a native oxide layer over the membrane surfaces. On the other hand, the standard membranes have a higher charge concentration per unit volume, which has a stimulating effect in osteoprogenitor cells and is in agreement with that expressed by Chiarenza, A.R. and Weiss, C.M. (1979) [36].

The biopsies done before the beginning of the study were collected to know the state of the bone remodelling at the sites close to the membrane implantation. Only osteocytes were observed in all of the cases that were studied. Over the external membrane surface and in the contact edges, the osteoblastic activity with new bone formation was observed in all of the cases. On the other hand, the results of the histologic analysis of the number of osteoblasts does not show a pattern throughout the study. Although at 20 days after surgery a higher number of osteoblasts was observed in comparison with the osteocytes in the tibia with the standard membrane, in the tibia with the high membrane the opposite was observed. Moreover, during the first days, there was a large number of chondroblasts, especially in the bone augments formed at 20 and 30 days after surgery, congruent with the longitudinal study design, which allowed us to justify the presence of cartilaginous tissue in the early stages of the development of new bone. Chondroblasts were located towards the top of the augmentation, showing the direction of the bone tissue formation and apposition. Under the chondroblasts, the osteoblasts were observed, indicating the direction of bone formation. Additionally, osteocytes were observed in the extracellular matrix (ECM) forming a reticular tissue, which coincides with results the expressed by Park, J. and Lakes, R. S. (2007) [37], who established that an increase in the amount of collagen marks the beginning of the formation process and occurs approximately after one week. At four weeks from the beginning, the bone callus is formed by three parts [38]. As can be observed in Figure 3c,d, the disposition of the trabeculae in the woven bone determines the appositional growth, agreeing with the density values over the timecourse for the tibiae with the standard membrane.

As described by Degidi, M. et al. [38], for titanium, the physical dimensions and disposition of the membrane over the subjacent bone influence the new bone formation. The Cr-Co-Mo alloy (ASTM F75) provides a scaffolding with an adequate microenvironment for vertical bone volume augmentation and prevents fibrous tissue invasion. Additionally, these non-resorbable membranes provide excellent mechanical and biocompatible properties to meet the needs of potential applications in guided bone regeneration. The longitudinal study achieved positive results that allow us to understand the early stages of bone formation, although additional studies with a large number of samples would be needed in order to statistically validate the results.

## 4. Materials and Methods

### 4.1. Cr-Co-Mo Membranes Manufacture

**Figure 5:**
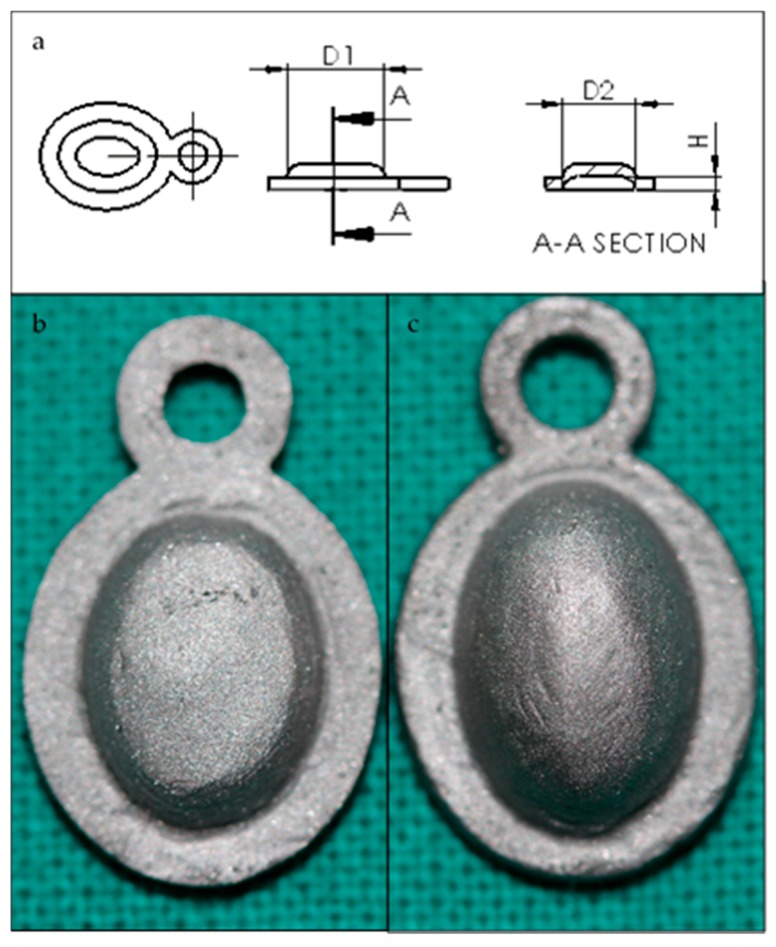
(**a**) Membrane prototype plan. D1 = 8 mm, D2 = 6 mm. (**b**) H = 1.2 mm (standard membrane) or (**c**) H = 2.4 mm (high membrane).

**Figure 6:**
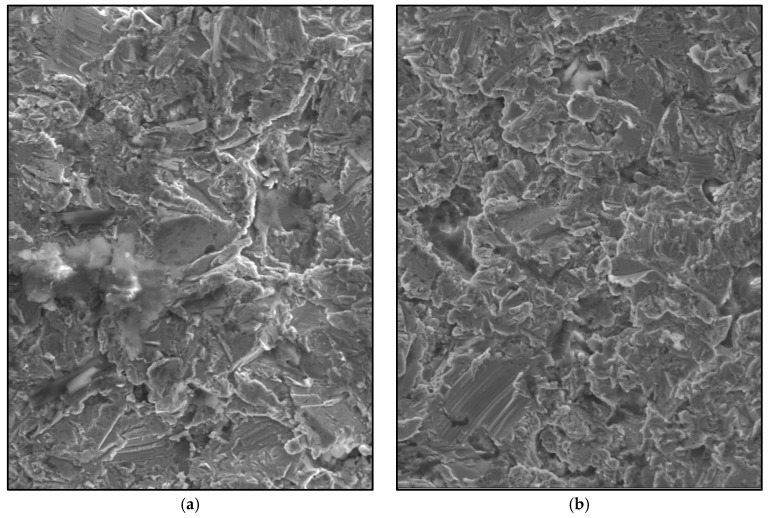
Scanning electron microscope (SEM) image of the sandblasted Cr-Co-Mo surface membranes (700×). (**a**) Standard membrane. (**b**) High membrane.

The membrane design was created using SolidWorks 2013 CAD software (Dassault Systems SolidWorks Corp, Concord, MA, USA) to grant uniformity to compare the achieved results. Prototypes were obtained (standard and augmented, Figure 5a) through 3D printing techniques with white opaque photopolymer (Vero White Plus^®^) with a 16-micrometre resolution. Prototypes were used as a mould to manufacture the membrane cast with the Cr-Co-Mo alloy (VITAL^®^), of Cr 28%, Mo 6%, and Co. 62% (Figure 5b). Both of the groups of membranes were sandblasted (Figure 6) with 180-µm aluminium oxide particles (Al_2_O_3_) with the same protocol. Membranes were cleaned in an ultrasonic bath of deionized water for 10 min before sterilisation.

### 4.2. Surgical Procedure

Approval from the Superior Council of the National University of Entre Rios was obtained prior to the start of the study, under the code 6160 through Res CS 237-15. Five adult New Zealand rabbits weighing between 2.8 kg and 3.4 kg were used as experimental animals. The animals were housed individually at 21.55 °C, with an average relative humidity of 68.37%. They were fed a standard commercial rabbit chow. Water and food were available ad libitum. The health status of the animals was regularly checked during the study.

Four experimental rabbits were divided in two groups: Group 1 (*n* = 4 right tibia of each animal) were implanted with standard membranes. Group 2 (*n* = 4 left tibia of each animal) were implanted with high membranes that provided a larger inner space for bone formation. Membranes were microfixed in order to avoid displacement or detachment. The remaining rabbit was the control.

Ketamine 50 (Holliday-Scott S.A^®^, Buenos Aires, Argentina) was administered intramuscularly for general anaesthesia, 1.5 mL of xylazine 100 (Richmond, Vet Pharma^®^, Buenos Aires, Argentina) was used as a muscle relaxant and 1.5 mL of Carticain L-adrenalin (Totalcaina Forte^®^, Laboratorios Bernabó, Argentina) was used as local anaesthesia. Radiographs of both tibiae were obtained on day 0 (before surgery). The surgical sites were shaved and disinfected with a povidone-iodine solution before the operation. A full thickness skin incision was made at the level of the proximal metaphysis to expose the tibia. The periosteum was removed from the bone surface, and then this area was covered with the membrane. A microperforation was done with a 1-mm diameter drill to attach the membrane with a vitallium microscrew, and the membrane completely touched the bone underneath. In the right tibiae, standard Cr-Co-Mo membranes were implanted using the same protocol; in the left tibiae, high membranes were implanted. In the control rabbit, the same surgical procedure was done, but a non-critical bone defect was made with a 3-mm diameter trephine, and no membrane was placed. The surgical wound was sutured closed with Nylon 3.0 (Ethilon, Dilbeek, Belgium), and antibiotics were administered. The sutures were removed after two weeks.

Biopsies were obtained from all of the extremities with a 3-mm diameter trephine close to the membrane implantation site to analyse the cellular components of the bone surface before membrane placement.

The animals were sacrificed at 20, 30, 40, and 60 days postoperatively, with an overdose of sodium pentobarbital (Dolethals; Vetoquinol, Lure, Saint Anne, Alderney, France).

### 4.3. Analysis

A computerized axial tomography analysis (TAC, Multidetector CT Scanner Philips Brilliance^®^ of 64 channels) was performed. The augmented bone volumes were calculated from those images. In addition, the mean density per unit area of the new bone was calculated.

**Figure 7:**
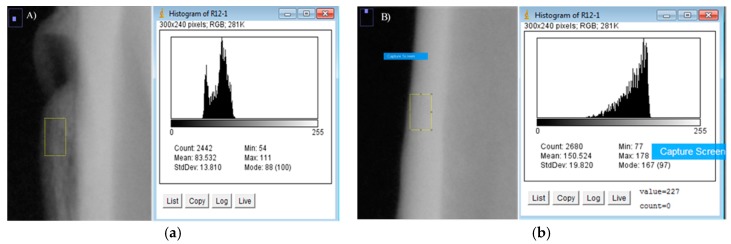
(**a**) Calculation of ROIint. (**b**) Calculation of ROIref.

For the densitometry analysis, a dental radiology apparatus (Carestream Health, Inc., 150, Verona Street, Rochester, New York, NY, USA) was used to obtain density values of the new bone. This information was then processed by an image analyser (ImageJ, NIH, Bethesda, ML, USA). The distribution of density was calculated by comparing the grey levels of each image. The selected areas were the augmented bone, used as a region of interest (ROIint, Figure 7a), and the cortical bone before the membrane implantation, used as region of reference (ROIref, Figure 7b), with a size selection window of 0.07 mm × 0.12 mm. Three measurements at different sites within the augmented volume were taken and were presented as average ± standard deviation. Densitometric studies were performed before the surgery and after the sacrifice of each animal.

The samples were fixed in a 10% formaldehyde solution for further analysis. Tissue samples containing the newly formed bone were removed for routine laboratory processing for decalcified sections placed in paraffin. The blocks were cut and stained with haematoxylin and eosin. Histologic analysis results are expressed as the mean number of osteocytes and osteoblasts that were counted in 10 microscope fields at a magnification of 40×.

## Figures and Tables

**Table 1:** Augmentation volumes obtained.

Days	Standard Membrane	High Membrane
	Augmentation Volume [mm^3^]	Augmentation Volume [mm^3^]
20	15.63	18.23
30	20.61	20.66
40	22.79	17.58
60	27.16	11.41

**Table 2:** Cell counting from the newly formed tissue.

Days	Average Osteoblasts		Average Osteocytes	
	Standard Membrane	High Membrane	Standard Membrane	High Membrane
20	960 (10 fgm)	310 (10 fgm)	580 (10 fgm)	940 (10 fgm)
30	50 (4 fgm)	1400 (10 fgm)	270 (4 fgm)	1100 (10 fgm)
40	1000 (10 fgm)	300 (10 fgm)	680 (10 fgm)	400 (10 fgm)
60	33 (10 fgm)	310 (10 fgm)	360 (10 fgm)	800 (10 fgm)

fgm: Fields of greatest magnification.

**Table 3:** Bone density values.

Days	Standard Membrane			High Membrane		
	ROIint (SD)	ROIref (SD)	Ratio (%)	ROIint (SD)	ROIref (SD)	Ratio
20	77.90 (0.85)	190.61 (8.64)	40.91 (1.61)	65.86 (2.29)	148.48 (4.91)	44.42 (3.01)
30	59.66 (5.69)	127.51 (20.99)	47.16 (3.92)	64.92 (5.13)	162.51 (3.33)	39.99 (3.76)
40	78.68 (8.18)	165.38 (3.09)	47.62 (5.57)	117.65 (13.74)	185.86 (3.82)	63.24 (6.64)
60	86.04 (1.80)	163.12 (13.55)	52.93 (3.28)	106.39 (4.29)	185.77 (1.17)	57.28 (2.58)
Control	153.46 (0.97)	162.94 (1.59)	94.19 (1.51)	153.97 (0.57)	163.88 (2.05)	93.96 (1.45)
